# Supplementary material for: A too-good-to-be-true prior to reduce shortcut reliance
Source: Pattern Recognit Lett. 2023 Feb;166:164–71. doi: 10.1016/j.patrec.2022.12.010 (PMC10615835; doi:10.1016/j.patrec.2022.12.010)
Supplement: Supplementary Data S1 — Supplementary Raw Research Data. This is open data under the CC BY license http://creativecommons.org/licenses/by/4.0/ [file mmc1.pdf]

Appendix A. Results for VGG-11 as the high-capacity network

Appendix A.1. Separability of shortcut and regular items in LCN- and HCN-IWs

As described for ResNet-56 in the main text, we used logistic regression to classify IWs as shortcut or regular. When the shortcuts in the training set were local, classification accuracy averaged across 45 class-pairs was 91.53% for LCN-IWs and 70.011% for HCN-IWs, with corresponding 95% CIs of [82.60, 93.45] and [70.01, 70.02]. When the shortcuts were global, average classification accuracy was 90.4% for LCN-IWs and 70.011% for HCN-IWs, with corresponding 95% CIs of [86.16, 94.66] and [70.010, 70.024]. These results repeat the pattern we observed for ResNet-56, confirming that LCN-IWs, compared to HCN-IWs, much better distinguish shortcut images from regular ones.

Appendix A.2. Test accuracies and overall benefit

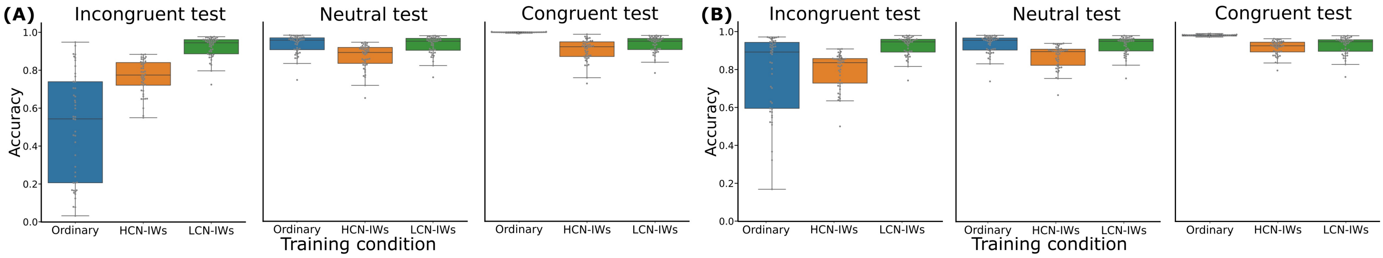

Fig. A.1: Accuracies on incongruent, neutral, and congruent test sets after ordinary and HCN-/LCN-weighted training, with (A) local or (B) global shortcuts in training set. HCN is VGG-11. Across shortcut types, LCN-IWs result in almost identically high accuracy on all three test sets and thus, are successful in avoiding shortcut reliance. HCN-IWs constantly result in accuracies inferior to LCN-IWs; moreover, on neutral and congruent test sets, accuracies after HCN-weighted training are lower than after ordinary training. HCN-IWs, thus, are not as effective as LCN-IWs in avoiding shortcut reliance and also result in suppressing useful features. Together, these results indicate that the LCN-HCN two-stage approach is a valid representative of the too-good-to-be-true prior.

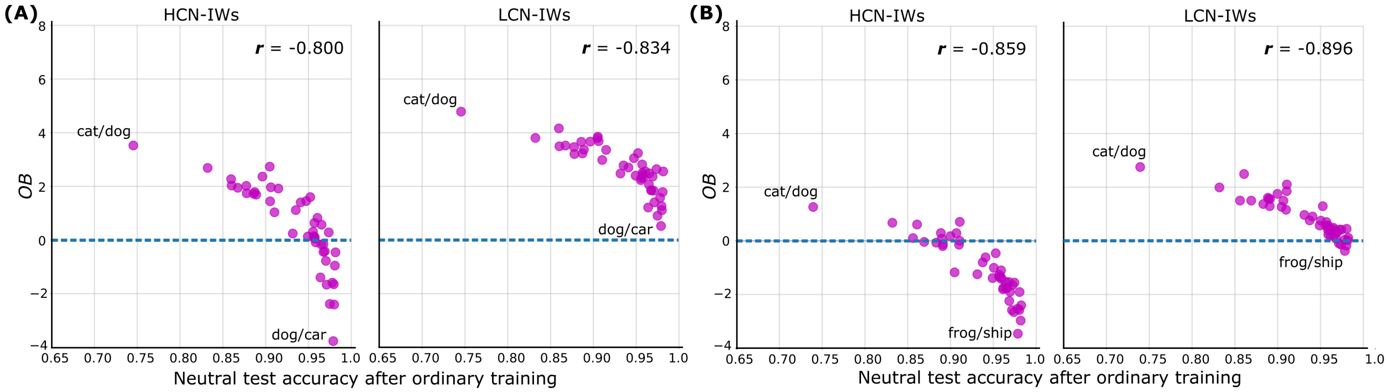

Fig. A.2: Effects of the LCN-/HCN-IWs training procedure for each of the 45 class pairs depending on a difficulty of the respective binary classification problem. HCN is VGG-11; (A) local and (B) global shortcuts are considered separately. Effects of training are represented by the Overall Benefit measure (*OB*; gain + loss; see main text, Section 3.2.4); the difficulty of a pair is represented by the neutral test accuracy after ordinary training on the training set. Recapitulating previous results, LCN-IWs are more effective than HCN-IWs. Furthermore, the easier learning problem, the less *OB* from IWs because the relatively higher capacity of a producing IWs network leads to downweighting non-shortcut items.

Appendix B. Additional illustrations of differences between LCN-IWs and HCN-IWs

Appendix B.1. Illustrative histograms of importance weights for a single pair of classes

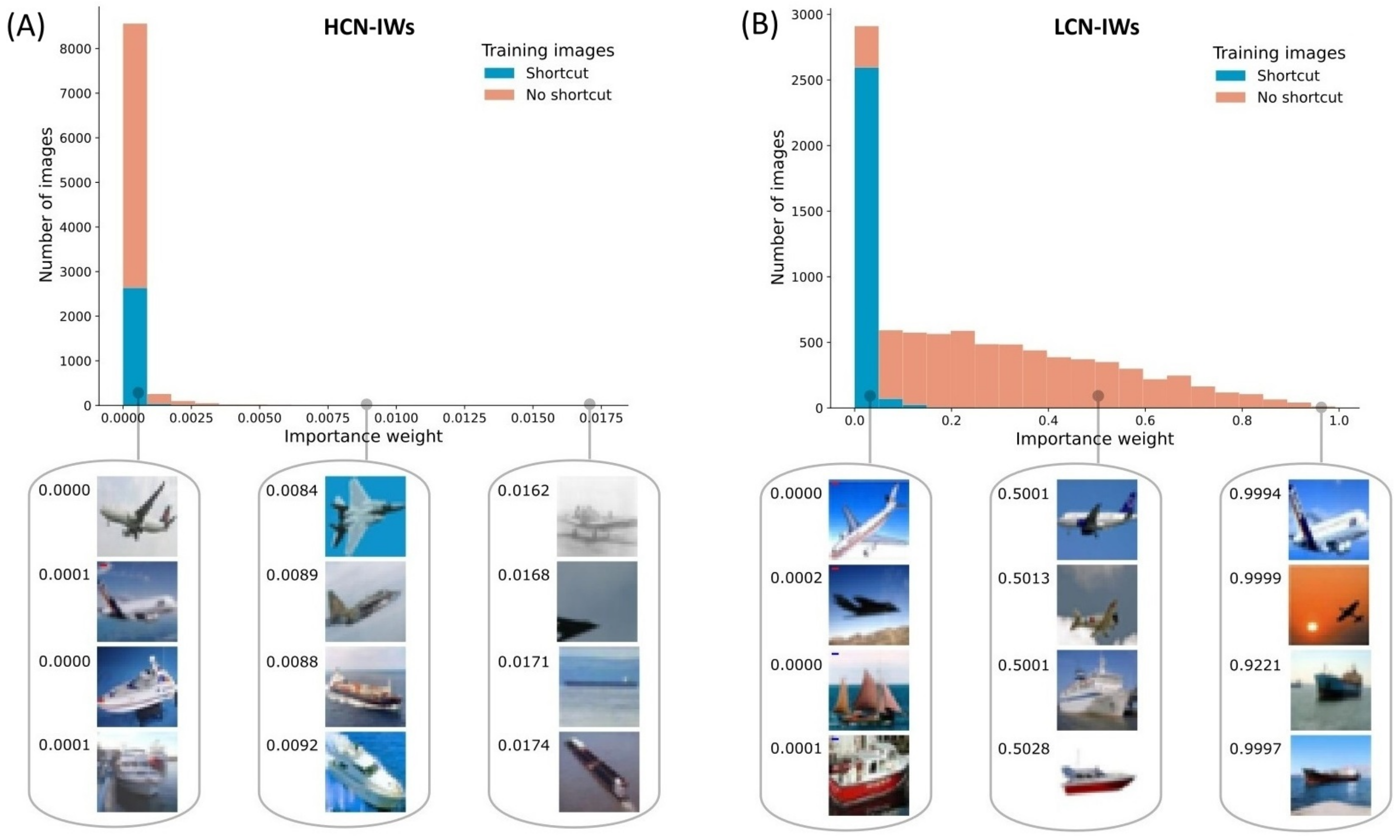

Fig. B.1: Observed distributions of HCN-IWs (A) and LCN-IWs (B) for the plane/ship pair with the local shortcut in the training set. HCN is ResNet-56. The near zero importance weights from the LCN were mostly for shortcut images. As predicted, the LCN has the capacity to master images containing shortcuts but few other images, providing IWs for an HCN that reduce shortcut reliance, thereby implementing the too-good-to-be true prior.

Appendix B.2. LCN-IWs and HCN-IWs correlation

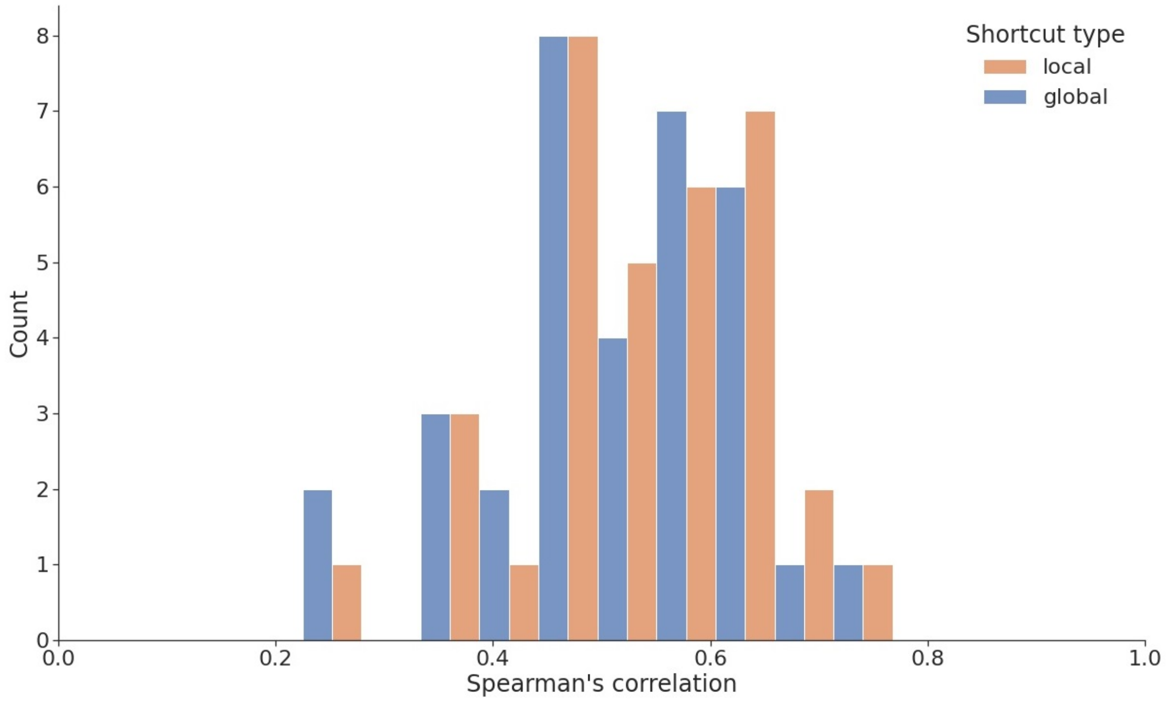

Fig. B.2: Spearman's rank correlations (r_S) between LCN-IWs and HCN-IWs: distribution of r_S across 45 pairs of classes (i.e., binary classification problems). All correlations are positive, and, for the majority of problems, are moderate.
